# Correlation between the native lung volume change and postoperative pulmonary function after single lung transplantation for lymphangioleiomyomatosis: Evaluation of lung volume by three-dimensional computed tomography volumetry

**DOI:** 10.1371/journal.pone.0210975

**Published:** 2019-02-11

**Authors:** Hirotoshi Suzuki, Hisashi Oishi, Masafumi Noda, Tatsuaki Watanabe, Yasushi Matsuda, Junya Tominaga, Tetsu Sado, Akira Sakurada, Hajime Kurosawa, Kei Takase, Yoshinori Okada

**Affiliations:** 1 Department of Thoracic Surgery, Institute of Development, Aging and Cancer, Tohoku University, Sendai, Japan; 2 Department of Diagnostic Radiology, Tohoku University Hospital, Sendai, Japan; 3 Department of Occupational Health, Tohoku University Graduate School of Medicine, Sendai, Japan; Massachusetts General Hospital, UNITED STATES

## Abstract

**Purpose:**

Whereas native lung overinflation has been thought to happen in recipients of single lung transplantation for lymphangioleiomyomatosis because of its increased compliance, there is no study that has reported the details on the change of the native lung volume after single lung transplantation by three-dimensional computed tomography volumetry. The purpose of the present study was to evaluate the lung volume after single lung transplantation for lymphangioleiomyomatosis by three-dimensional computed tomography volumetry and investigate the correlation between the native lung volume change and postoperative pulmonary function.

**Methods:**

We retrospectively reviewed the data of 17 patients who underwent single lung transplantation for lymphangioleiomyomatosis. We defined the ratio of the native lung volume to total lung volume (N/T ratio) as an indicator of overinflation of the native lung. In order to assess changes in the N/T ratio over time, we calculated the rate of change in the N/T ratio which is standardized by the N/T ratio at 1 year after single lung transplantation: rate of change in N/T ratio (%) = {(N/T ratio at a certain year)/(N/T ratio at 1 year)– 1}× 100.

**Results:**

We investigated the correlations between the N/T ratio and the pulmonary function test parameters at 1 year and 5 years; however, there was no significant correlation between them. On the other hand, there was a significant negative correlation between the rate of change in the N/T ratio and that in forced expiratory volume in 1 second %predicted (%FEV1) at 5 years after single lung transplantation.

**Conclusion:**

The single lung transplantation recipients for lymphangioleiomyomatosis showed increased rate of change in the N/T ratio in the long-time course after lung transplantation with the decrease of %FEV1. We expect that these cases will probably cause the overinflation of the native lung in the future.

## Introduction

Lung transplantation is an established therapeutic option for patients with various types of end-stage lung disease. Internationally, the annual number of bilateral lung transplants (BLTs) is greater than that of single lung transplants (SLTs) because of the better survival with the former procedure [1]. On the other hand, the current situation in Japan is completely different from other countries. The number of SLTs performed in Japan is almost the same as the number of BLTs. Miyoshi et al. reported that, given the severe donor shortage in Japan, SLT can be the first choice of surgical procedure type with acceptable outcomes if there is no contraindication to SLT [2].

The Toronto lung transplant group conducted cadaveric SLT for a patient with pulmonary fibrosis and achieved long-term survival as the first successful lung transplantation case in the world in 1983 [3]. Mal et al. successfully performed cadaveric SLT for a patient with chronic obstructive pulmonary disease (COPD)[4]. COPD had been considered as an indication for SLT after this successful case. However, overinflation started to be recognized as one of the most serious complications after SLT for COPD. In SLT recipients for COPD, the overinflation of the native lung is thought to occur due to increased compliance, whereas the transplanted lung graft on the other side has normal compliance. Pneumonectomy of the native lung [5], lung volume reduction surgery (LVRS) [6], and bronchoscopic lung volume reduction (BLVR) [7] were reported as therapeutic modalities for overinflation of the native lung.

Lymphangioleiomyomatosis (LAM) is a rare cystic lung disease that develops primarily in women of childbearing age and is characterized by the proliferation of abnormal smooth muscle-like cells (LAM cells). LAM is associated with an obstructive disease pattern and reduced diffusion capacity of the lung for carbon monoxide (DLco) determined by a pulmonary function test, the main reason for which is the increased lung compliance [8]. Due to the different compliance between the native and the transplanted lungs, the native lung overinflation can be a serious complication after SLT for LAM, that is likely to occur after SLT for COPD. Liu et al. reported a case of pneumonectomy of an overinflated native lung after SLT for LAM that resulted in improved lung function [9].

CT scans and chest X-ray often show the mediastinum shifted to the transplanted lung side in recipients of SLT for LAM. Whereas native lung overinflation has been thought to happen in recipients of SLT for LAM because of its increased compliance, there is no study that has reported the details on the change of the native lung volume after SLT by three-dimensional computed tomography (3D-CT) volumetry. One of the advantages of 3D-CT volumetry is that we can quantitatively analyze the lung volume in a non-invasive manner and determine changes in the lung over time.

We observe that some recipients of SLT for LAM deteriorate pulmonary function with findings of the mediastinum shifted to the transplanted lung side. It is speculated that the overinflation of the native lung affects the pulmonary function after SLT for LAM. However, there is currently no established way to objectively diagnose the overinflation of the native lung. The purpose of the present study was to evaluate the lung volume after SLT for LAM by 3D-CT volumetry and investigate the correlation between the native lung volume change and postoperative pulmonary function in the recipient.

## Patients and methods

### Patients

Between January 2006 and December 2015, 25 patients underwent SLT for LAM at Tohoku University Hospital. Eight of these patients were excluded from this study: 3 patients died of primary graft dysfunction in the perioperative period; 1 patients died of enteritis at 6 months after SLT; 1 patients developed deformation of the thorax and received retransplantation within 4 years after SLT; 3 patients developed chronic lung allograft dysfunction (CLAD) of, at least, BOS 0-p.[10] Thus, we analyzed the results of 17 patients who had undergone SLT and survived for more than 3 years. Pulmonary function test (PFT) data was available in 17 patients at post-transplant 1 year, 17 patients at 2 post-transplant years, 17 patients at 3 post-transplant years, 15 patients at post-transplant 4 years and 10 patients at post-transplant 5 years. In 9 patients, PFT data and low attenuation volume (LAV) analysis (described later) were available every year up to post-transplant 5 years. The Institutional Review Board of Tohoku University Hospital approved the study (approval No. 2017-1-021) and research was conducted in accordance with the 2000 Declaration of Helsinki. Written informed consent have been obtained from all participants.

### Image acquisition and measurement of lung volume by three-dimensional computed tomography volumetry

**Fig 1:**
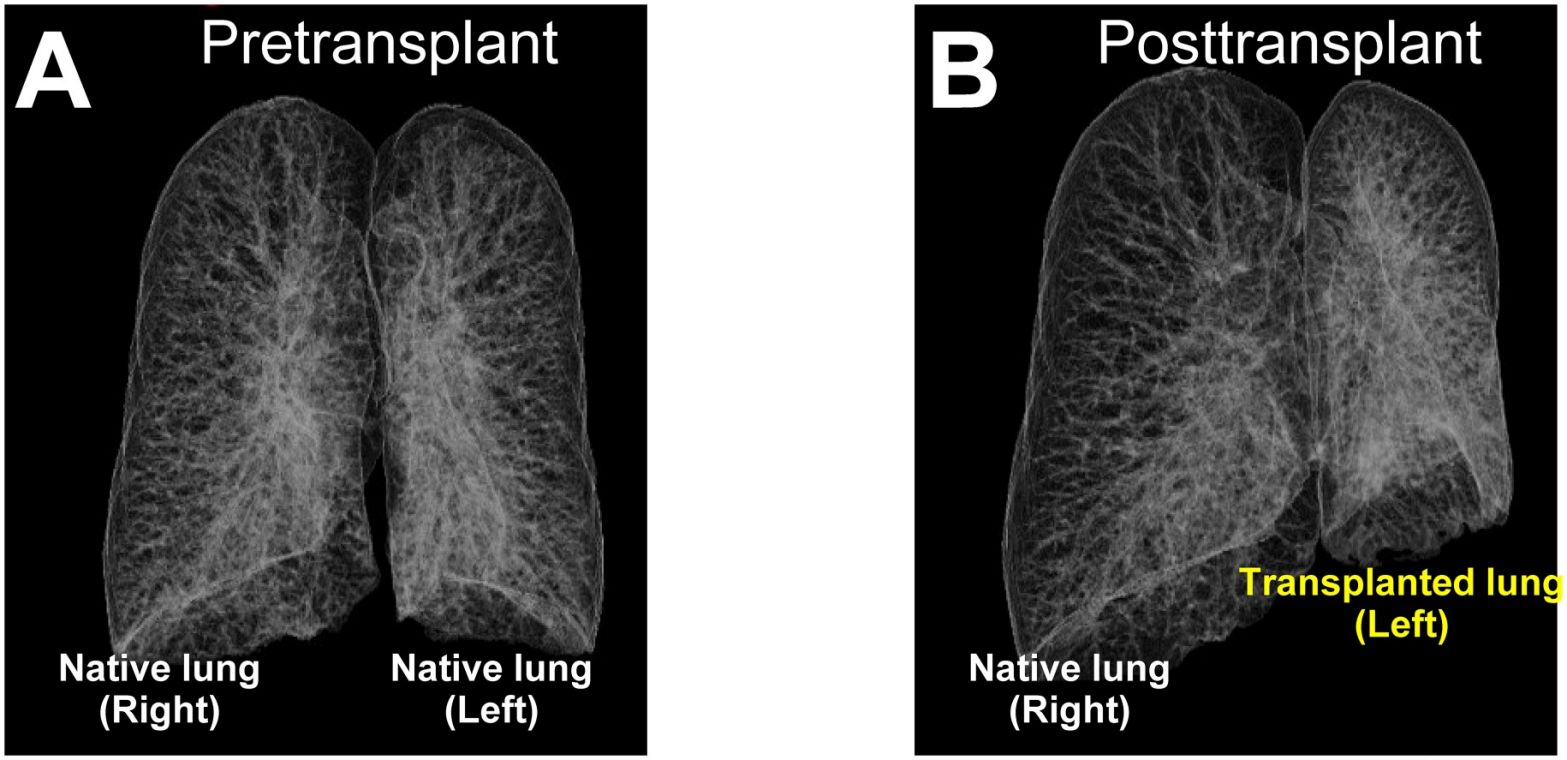
Pre- and post-transplant three-dimensional computed tomography (3D-CT) images of a case of single lung transplantation for lymphangioleiomyomatosis. (A) Pretransplant three-dimensional computed tomography (3D-CT) image of the lungs in a case of lymphangioleiomyomatosis. (B) Post-transplant 3D-CT images of the lungs in the case: a left single lung transplant case.

CT images were obtained during a single respiratory pause at the end of maximum inspiratory effort using a multidetector row CT scanner (BrightSpeed Elite, GE Healthcare Japan Ltd, Tokyo, Japan). Whole lung scans were performed at a peak tube voltage of 120 kVp, with a variable mAs setting using an automatic exposure control system. CT data from 1-2-mm slices were used for the volumetric analysis. Each patient’s CT data were transferred to a standalone workstation (Ziostation2, Ziosoft, Inc., Tokyo, Japan) and 3D models were reconstructed. Left and right lung volumes were calculated by summing the voxels of the CT value of the window level (WL), −469 Hounsfield units (HU); window width (WW), 684.8 HU; sharpness (SH), 0. The respiratory tract from the trachea to subsegmental bronchi was reconstructed and automatically excluded from the lung volumes. Fig 1 shows pre- and post-transplant 3D-CT images of a case of SLT for LAM. We manually measured the lung volumes with the same values of WL, WW, and SH when they could not be measured automatically. In the case of manual measuring, two radiologists measured the lung volumes in a blinded manner and the mean value was used for the study. The data of PFT and 3D-CT volumetry at each time point after SLT were collected.

### Evaluation of overinflation of the native lung

Currently, there is no standard method to evaluate the degree of overinflation of the native lung after SLT. We defined the ratio of the native lung volume to total lung volume (N/T ratio) as an indicator of overinflation of the native lung. The N/T ratio was calculated as follows:

N/Tratio=nativelungvolume/totallungvolume×100(%)

In order to assess changes of the N/T ratio over time, we calculated the rate of change in the N/T ratio, which is standardized by the N/T ratio at 1 year after SLT. In the present study, we call this parameter, the ‘rate of change in N/T ratio’ which was calculated as follows:

RateofchangeinN/Tratio(%)={(N/TratioatacertainyearafterSLT)/(N/Tratioat1yearafterSLT)–1}×100.

Rate of change in forced expiratory volume in 1 second %predicted (%FEV1) was also calculated in the same way when we investigated the correlation between the rate of change in N/T and %FEV1.

### Measurement of low attenuation volume: Evaluation of emphysematous lesions

LAV was calculated by summing the voxels with CT values of less than –950 HU. LAV was reported to be the volume of emphysematous lesions in COPD patients[11]. The percentage of LAV to total lung volume is considered to reflect the severity of COPD of the lung [11]. In the present study, in order to evaluate emphysematous lesions in the native lung, the percentage of LAV in the native lung was calculated as follows:

NativelungLAV%=nativelungLAV/nativelungvolume×100.

RateofchangeinnativelungLAV%(%)={(nativelungLAV%atacertainyearafterSLT)/(nativelungLAV%at1yearafterSLT)–1}×100.

### Evaluation of size matching

Under the current lung allocation system in Japan, lungs are allocated in accordance with size matching, which is calculated using predicted vital capacities (pred VCs) of the donor and the recipient. Pred VC and size matching were calculated as follows:

PredVCformale(L)=0.045×height(cm)-0.023×age-2.258

PredVCforfemale(L)=0.032×height(cm)-0.018×age-1.178

Sizematching(%)=predVCofdonor/predVCofrecipient×100.

### Postoperative assessment of pulmonary function

Postoperative PFTs were performed using CHESTAC-8800 or 8900 (Chest Ltd., Tokyo, Japan) to measure VC, forced vital capacity (FVC), forced expiratory volume in 1 second (FEV1), DLco, and the DLco/alveolar volume (VA). The 6-minute walking distance (6MWD) (m) was measured according to the American Thoracic Society guidelines. Arterial blood gas (ABG) analysis was performed by RADIOMETER ABL 800 FLEX. Alveolar—arterial gradient (A-aDO_2_) was calculated as follows:

$$\text{A}-{\text{aDO}}_{2}=713\times 0.21-{\text{PaCO}}_{2}/0.8-{\text{PaO}}_{2}.$$

### Statistical analysis

All statistical analyses were performed using JMP, version 13, for MAC (SAS Institute Inc.; Cary, NC). All data are presented as the means ± SD or numbers of patients. The differences between groups were analyzed using Student’s *t*-test for continuous variables. We used ANOVA for multi-group analysis and the paired t-test to analyze paired groups. Linea regression was used to describe associations between selected parameters. *P* < 0.05 was considered significant.

## Results

### Recipient and donor characteristics and size matching of the lung

Table 1 shows characteristics of the donors and the recipients, and the size matching of the lungs. As mentioned in methods, the recipients were allocated the lungs in accordance with size matching; therefore, height and age were similar between the recipients and the donors.

**Table 1:** Recipient and donor characteristics and size matching of the lung.

Variable	Donor (n = 17)	Recipient (n = 17)
Sex (male/female)	3 (17.6%)/14 (82.4%)	0/17
Height (cm)	156.0 ± 8.6	156.7 ± 5.6
Age	44.4 ± 12.6	46.6 ± 7.7
Predicted vital capacity (ml)*	2901.4 ± 496.4	2827.4 ± 212.9
Size matching of lung (%)^†^	108.2 ± 18.9 (87–147)	

Data are expressed as group mean ± standard deviation or number (%).

* Predicted vital capacity for male (L) = 0.045 × height (cm) − 0.023 × age − 2.258, predicted vital capacity for female (L) = 0.032 × height (cm) − 0.018 × age − 1.178.

^†^ Size matching (%) = predicted vital capacity of recipient / predicted vital capacity of donor × 100.

### Surgical and postoperative characteristics

Nine of 17 cases (52.9%) were right SLT. The duration of mechanical ventilation was 6.9 ± 6.5 days. The intensive care unit stay was 15.5 ± 9.6 days (S1 Table).

### Lung volume after SLT

**Fig 2:**
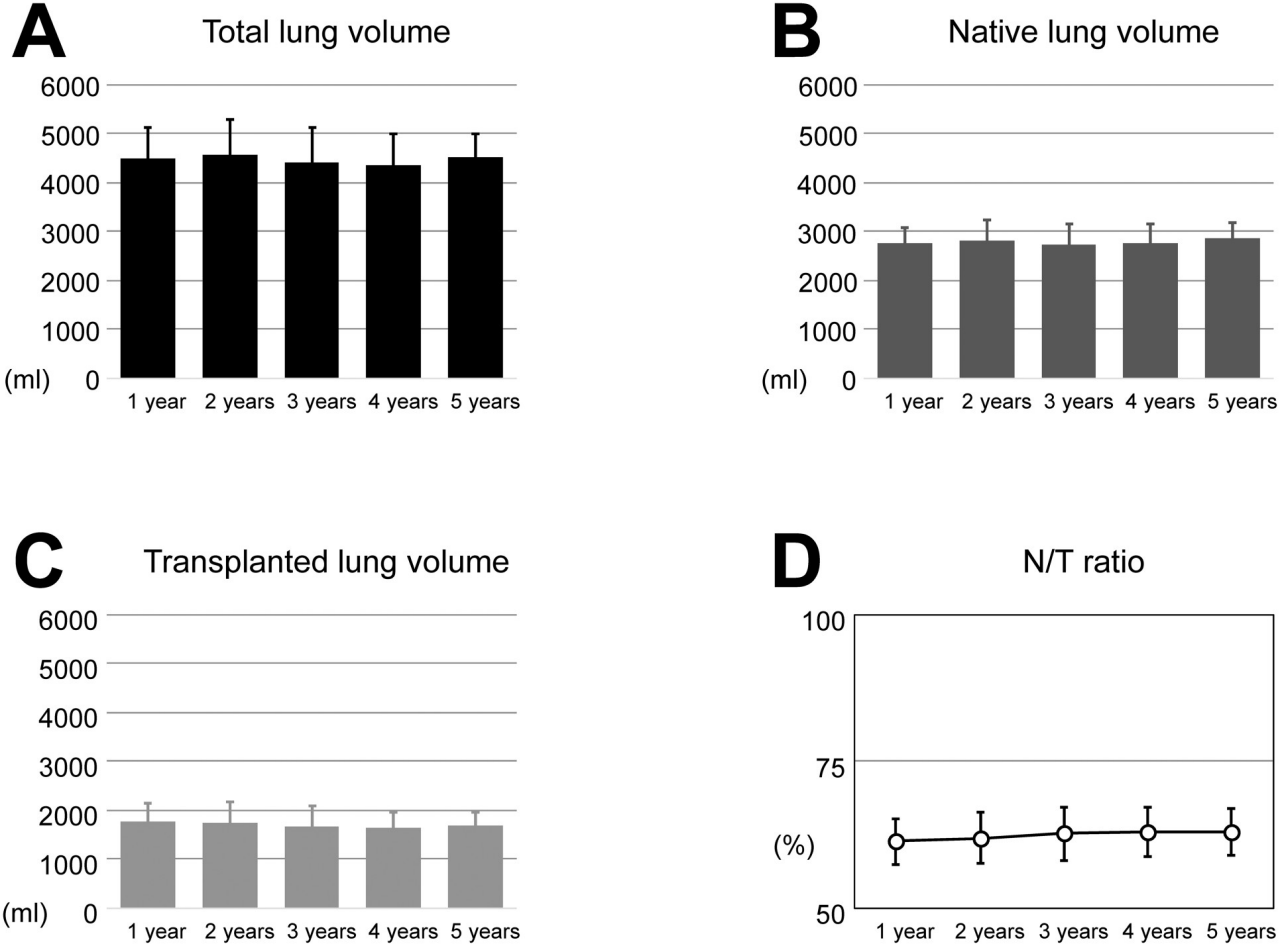
Lung volume after single lung transplantation. (A) The total lung volumes at 1, 2, 3, 4 and 5 years after single lung transplantation were 4495.2 ± 621.2, 4555.1 ± 724.4, 4391.7 ± 732.8, 4358.7±636.1, and 4521.8 ± 476.6 ml, respectively. There was no significant difference among the years. (B) The native lung volumes at 1, 2, 3, 4 and 5 years were 2742.3 ± 338.8, 2807.2 ± 411.7, 2733.7 ± 402.5, 2736.6 ± 419.1 and 2844.0 ± 337.0 ml, respectively. The native lung volumes showed a tendency to increase; however, they did not differ significantly among the years. (C) The transplanted lung volumes at 1, 2, 3, 4 and 5 years were 1752.9 ± 374.8, 1747.9 ± 420.3, 1658.1 ± 429.5, 1622.0 ± 326.7, and 1677.8 ± 270.7 ml, respectively. There was no significant difference among the years. (D) The ratios of the native lung volume to total lung volume (N/T ratios) at 1, 2, 3, 4 and 5 years were 61.3 ± 3.9, 61.9 ± 4.4, 62.6 ± 4.6, 62.9 ± 4.3 and 62.9 ± 4.0%, respectively. The N/T ratio showed a tendency to increase; however, they did not differ significantly among the years.

**Fig 3:**
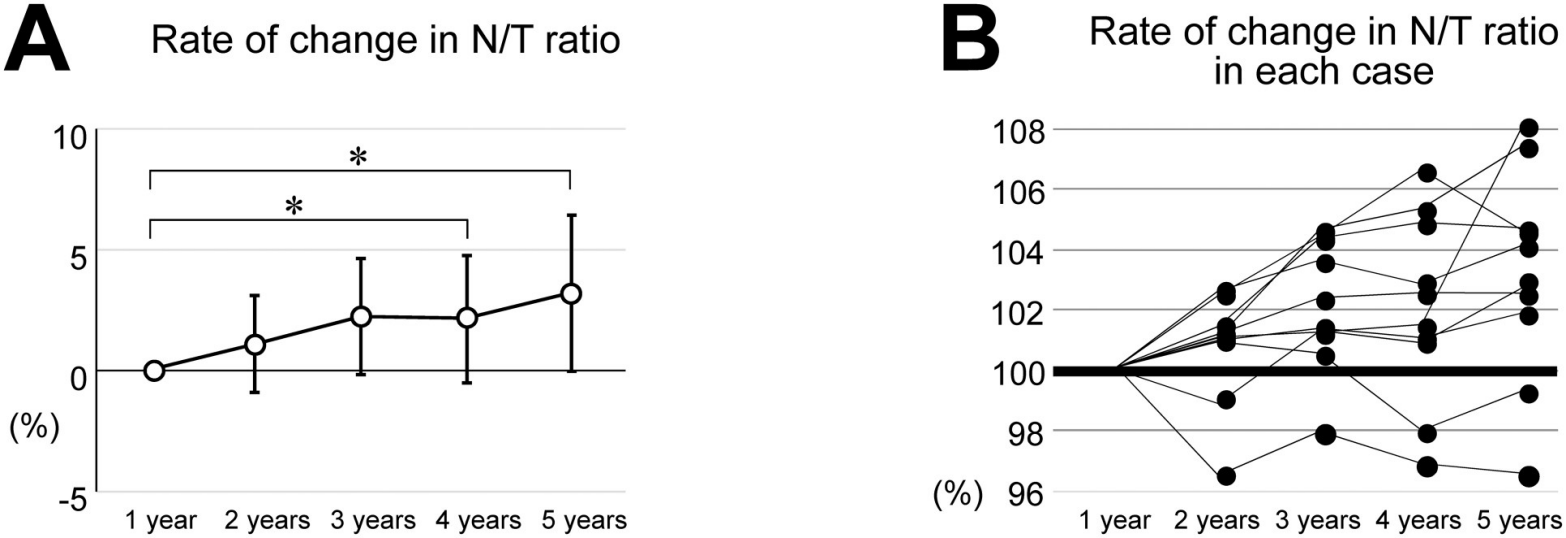
Rate of changes in the native lung volume to total lung volume (N/T ratio). (A) The rate of changes in the N/T at each year standardized by the N/T ratio at 1 year were 0, 1.0 ± 2.0, 2.2 ± 2.4, 2.1 ± 2.7 and 3.2 ± 3.2% (1, 2, 3, 4 and 5 years), respectively. The rate of change in the N/T ratio increased over time and reached a significant increase at 4 and 5 years after SLT. N/T ratio = the ratio of the native lung volume to total lung volume. (B) The rate of change in the N/T ratio in each case at 1, 2, 3, 4 and 5 years. Seven of 9 cases that we were able to follow the lung volume and pulmonary function test (PFT) data showed the increase of rate of change in the N/T ratio over year.

The total lung volume, the native lung volume and the transplanted lung volume measured by 3D-CT volumetry are shown in Fig 2A–2C. In 3 of 17 cases (17.6%), we manually measured the lung volumes because they could not be measured automatically. The native lung volume and N/T ratio showed a tendency to increase; however, they did not differ significantly among the years. The transplanted lung volume did not show any difference (Fig 2B and 2D). On the other hand, the rate of change in the N/T ratio increased over time and reached a significant increase at 4 and 5 years after SLT (Fig 3A). The rate of change in the N/T ratio in each case is presented in Fig 3B. Seven of 9 cases that we were able to follow the lung volume and PFT available every year showed the increase of the rate of change in the N/T ratio over year (Fig 3B).

### Native lung LAV, transplanted lung LAV and native lung LAV% after SLT

**Fig 4:**
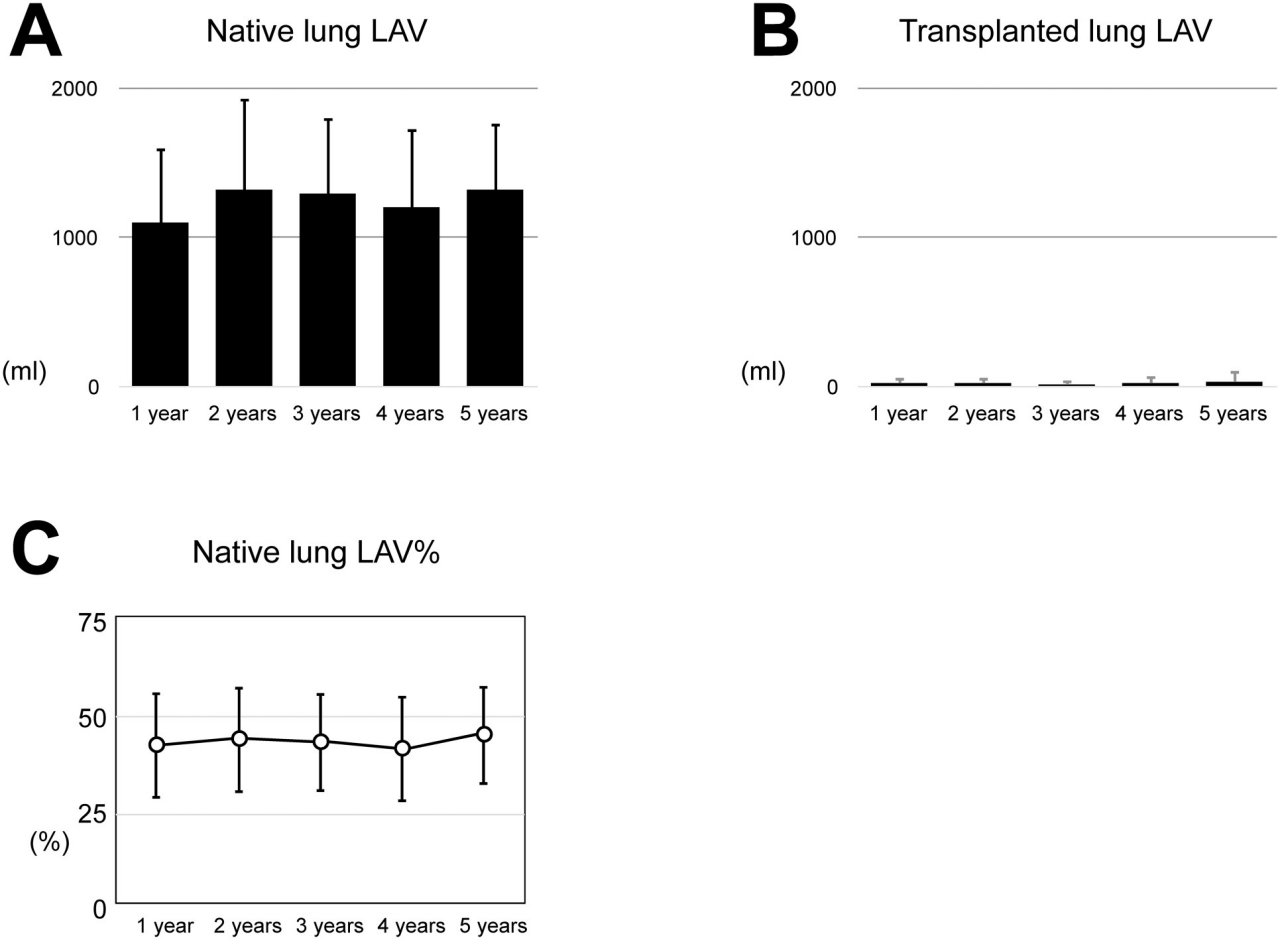
Low attenuation volumes of the native and transplanted lung. (A) The native lung low attenuation volumes (LAVs) at 1, 2, 3, 4 and 5 years were 1097 ± 493, 1320 ± 601, 1292 ± 497, 1203 ± 508 and 1321 ± 432 ml, respectively. There was no significant difference among the years. (B) The transplanted lung LAVs at 1, 2, 3, 4 and 5 years were 17.7± 26.2, 17.5 ± 33.4, 12.7 ± 18.7, 19.0 ± 33.8 and 29.4 ± 65.8 ml, respectively. There was no significant difference among the years. (C) The percentages of LAV in the native lung (native lung LAV%) at 1, 2, 3, 4 and 5 years were 42.1 ± 13.7, 43.2 ± 13.2, 41.9 ± 12.2, 39.7 ± 13.3 and 46.0 ± 12.7%, respectively. Native lung LAV% showed no significant change over time. Native lung LAV% = native lung LAV/native lung volume × 100.

**Fig 5:**
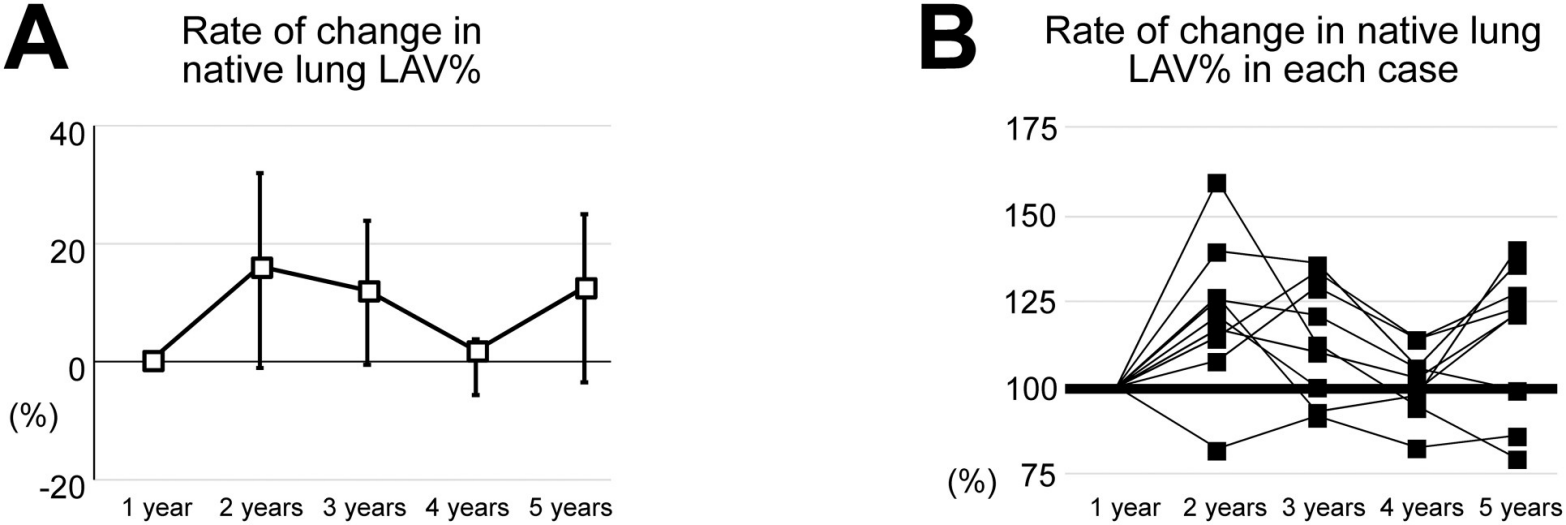
Rate of changes in low attenuation volumes of the native lung. (A) The rate of changes in the low attenuation volumes of the native lung (native lung LAV) at each year standardized by the native lung LAV at 1 year were 0, 17.8 ± 19.3, 13.3 ± 14.2, 1.9 ± 8.4 and 13.9 ± 18.1% (1, 2, 3, 4 and 5 years), respectively. (B) The rate of change in the native lung LAV in each case at 1, 2, 3, 4 and 5 years. Six of 9 cases showed the increase of rate of change in the native lung LAV% over year.

The native and the transplanted lung LAV showed no significant change among the years (Fig 4A). The transplanted lung LAV at each year was much smaller than the native lung LAV (Fig 4B). The native lung LAV% showed no significant change over time (Fig 4C). The rate of change in the native lung LAV% showed no significant change over time (Fig 5A). The rate of change in the native lung LAV% in each case is presented in Fig 5B. Six of 9 cases that we were able to follow LAV and PFT available every year showed the increase of rate of change in the native lung LAV% over year (Fig 5B). The 6 patients also showed the increase of rate of change in the N/T ratio over year.

### PFT, 6MWD and ABG after SLT

%FEV1 showed tendency to decrease; however, they did not differ significantly among the years. Other PFT parameters also showed no significant difference among the years (S2 Table). Similarly, 6MWD and all parameters of ABG analysis showed no significant difference among the years (S2 Table).

### Correlations between N/T ratio and PFT or ABG parameters or 6MWD after SLT

**Fig 6:**
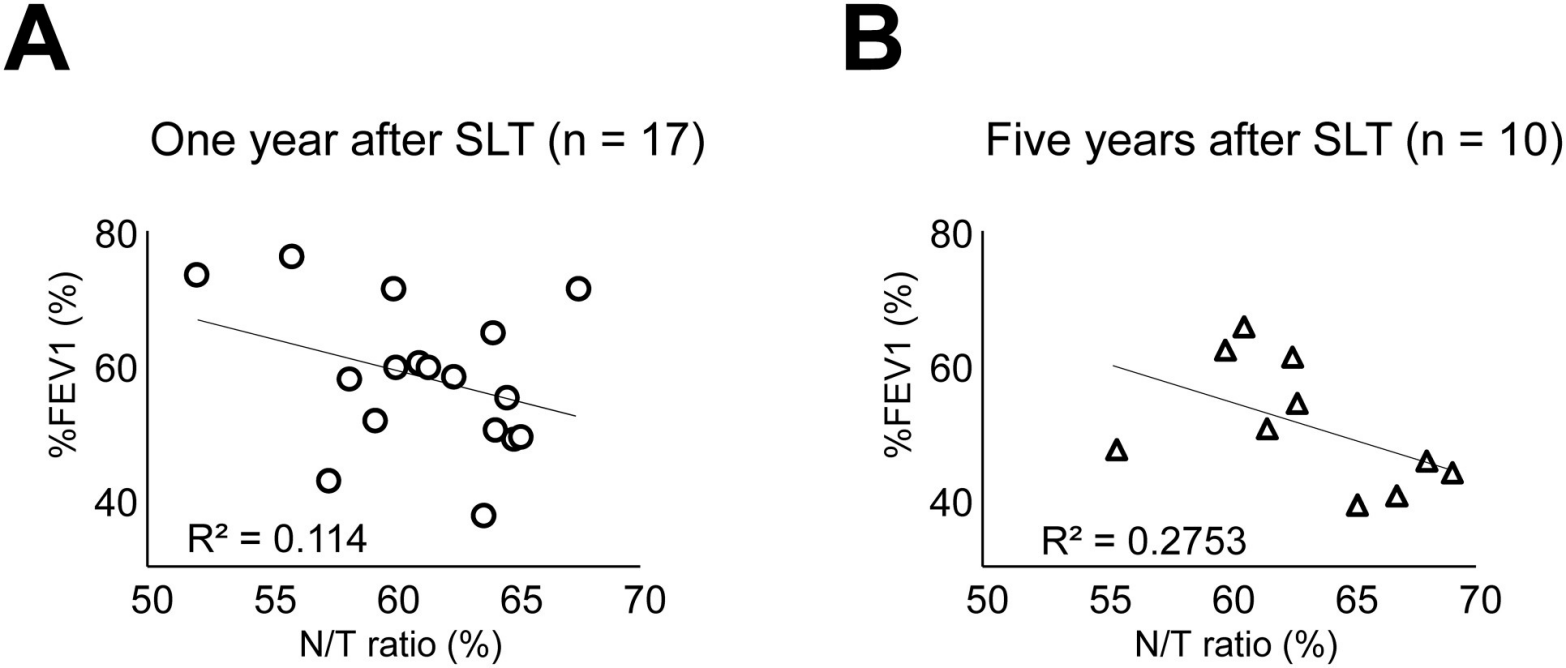
Correlations between native lung volume/total lung volume ratio and forced expiratory volume in 1 second %predicted. (A) There was no significant correlation between native lung volume/total lung volume (N/T) ratio and forced expiratory volume in 1 second %predicted (%FEV1) at 1 year after single lung transplantation (SLT). (B) N/T ratio did not show a significant correlation with %FEV1 at 5 years after SLT.

Fig 6A shows the correlation between N/T ratio and %FEV1 at 1 year after SLT. There was no significant correlation between them. We investigated the correlations between the N/T ratio and other PFT or ABG parameters or 6MWD at 1 year; however, there was no significant correlation between them (S1A, S2A and S3A Figs). Similarly, we examined the correlations between the N/T ratio and PFT or ABG parameters or 6MWD at 5 years after SLT. There was no significant correlation. (Fig 6B shows the correlations between the N/T ratio and %FEV1 at 5 years after SLT. The rest of the data at 5 years are shown in S1B, S2B and S3B Figs).

### Correlations between rate of change in N/T ratio and that in PFT parameters after SLT

**Fig 7:**
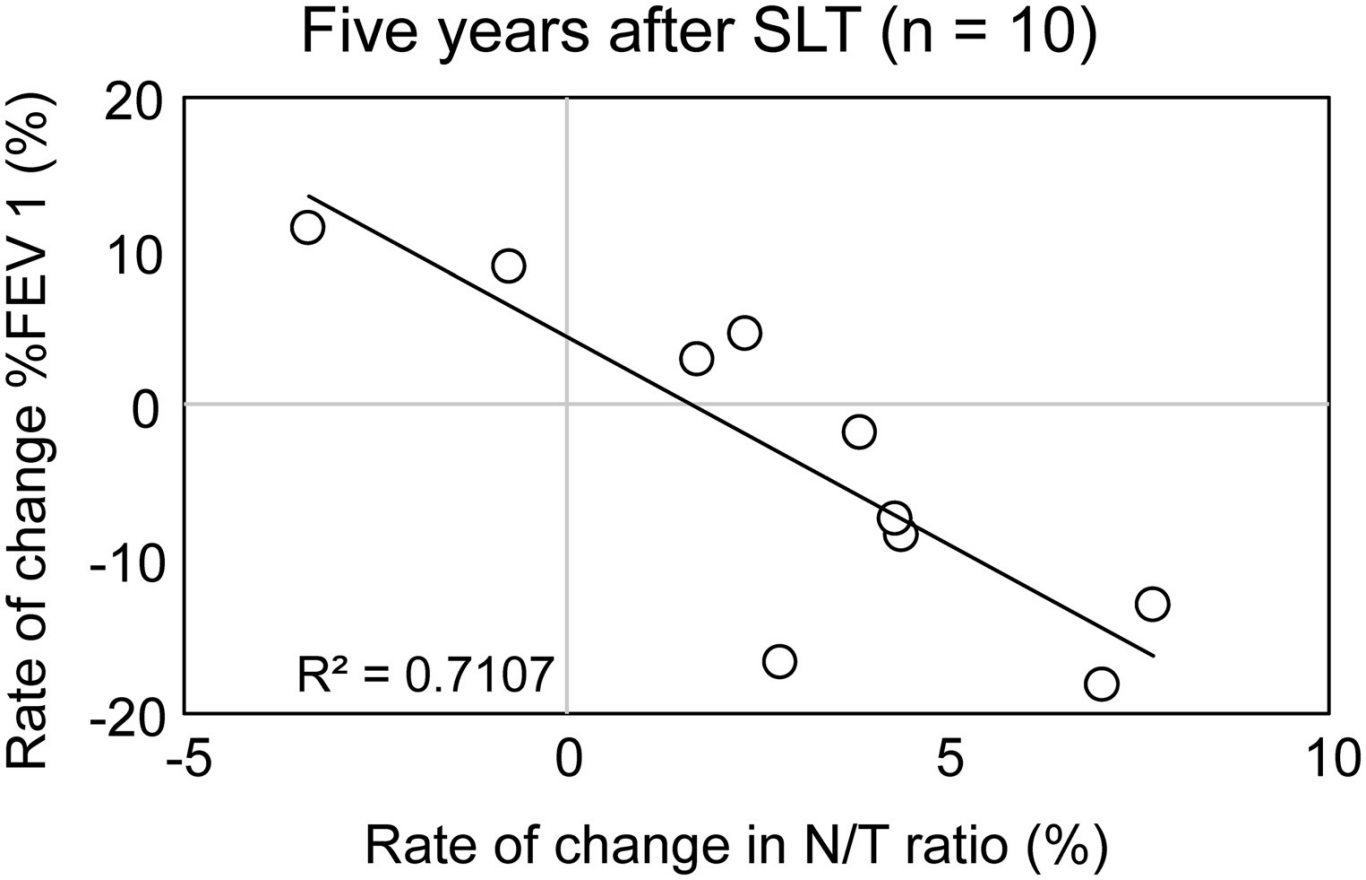
Correlations between rate of change in native lung volume/total lung volume ratio and change in forced expiratory volume in 1 second %predicted. There was a significant negative correlation between rate of change in native lung volume/total lung volume (N/T) ratio and that in forced expiratory volume in 1 second %predicted (%FEV1) at 5 year after single lung transplantation (SLT).

As shown in Fig 3A, the rate of change in the N/T ratio showed a significant increase over time. Therefore, we investigated correlations between the rate of change in the N/T ratio and that in PFT parameters at 5 years after SLT. There was a significant negative correlation between the rate of change in the N/T ratio and that in %FEV1 (Fig 7). The rate of change in the N/T did not show a significant correlation with the rate of change in other PFT parameters: %FVC, %DLco, DLco/VA (S4–S6 Figs).

### Correlations between rate of change in native lung LAV% and that in PFT parameters after SLT

We also investigated correlations between the rate of change in native lung LAV% and that in PFT parameters after SLT in the same way of change in N/T ratio mentioned above. There was no significant correlation between the rate of change in native lung LAV% and that in any PFT parameters (S7–S10 Figs).

## Discussion

Overinflation of the native lung is one of the challenging complications after SLT for obstructive lung diseases, such as COPD. In such patients, the compliance of the native lung is much higher than that of the healthy lung, transplanted lung. As a result of the difference in the compliance between the native and transplanted lungs, overinflation can occur only in the native lung. We recognize overinflation of the native lung after SLT in COPD by the shifted mediastinum in CT and chest X-ray. LVRS [6] and BLVR [7] have been applied for this complication in SLT recipients for COPD. Some SLT recipients for LAM also show a shift of the mediastinum to the transplanted lung side. However, it still remains unclear if overinflation of the native lung really happens in such patients. Another question we have in daily practice is whether overinflation of the native lung affects the post-transplant pulmonary function of the SLT recipients for LAM.

Nowadays, preoperative 3D-CT volumetry is utilized for lung cancer patients to predict the postoperative pulmonary function [12]. 3D-CT volumetry has also been commonly applied for the assessment of size matching in lung transplantation [13–15]. However, there is few studies that validated the usefulness of 3D-CT volumetry for the assessment of overinflation of the native lung after SLT.

In the present study, we found that, whereas the mean of the native lung volume did not show a significant increase, the rate of change in the N/T ratio significantly increased over time after SLT for LAM. Generally, pulmonary function stabilizes at 1 year after lung transplantation. It was reported that the lung function of recipients improved after SLT and then plateaued by 1 year post-transplantation [16]. Therefore, when we evaluated the rate of change of PFT parameters and N/T ratio, we standardized them by the values at 1 year after SLT. We demonstrated that 8 of 10 SLT recipients for LAM have increased the rate of change in the N/T over year that will probably cause the overinflation of the native lung in the future (Fig 7).

In order to assess the influence of the change in the N/T, we investigated the correlation between the N/T ratio and each PFT parameter at 1 and 5 years. We did not find any correlation between N/T ratio and each PFT parameter at either 1 or 5 years. On the other hand, there was a significant negative correlation between the rate of change in the N/T ratio and that in %FEV1 at 5 years after SLT. According to this result, if the N/T ratio during a long period—from postoperative 1 to 5 years—increases in a SLT recipient for LAM, %FEV1 will become worse. Indeed, Fig 7 shows that 6 recipients with a positive rate of change in N/T ratios showed negative a rate of change in %FEV1.

Whereas we revealed that the rate of change in the N/T affects the pulmonary function in recipients of SLTs for LAM, it remains unclear whether the overinflation of the native lung would really happen and finally cause end-stage respiratory failure in the future. We will have to follow up the recipients in the present study for a longer time in order to clarify wheter VRS or BLVR can be offered as an option for recipients with end-stage respiratory failure after SLT for LAM.

It has been reported that by evaluating the low attenuation area (LAA (%)), we can predict mortality [17] and exacerbations [18] of emphysema in COPD patients, and by evaluating LAV, we can differentiate subtypes of COPD [19] and predict the incidence of complications after surgery for lung cancer [11]. LAA is also utilized for differential diagnosis including LAM and other diseases [20]. We measured LAV from 1 to 5 years after SLT to evaluate the change in the cystic region. As shown in Fig 3A, LAV did not differ significantly from 1 to 5 years. This demonstrates that the cystic regions of LAM did not progress in the recipients in the present study and the decrease of %FEV1 was not associated with the progression of LAM.

This study has some limitations. First, the N/T ratio was originally defined in this study as an indicator of native lung overinflation. We are still not sure that this indicator really reflects the native lung overinflation. However, in previous reports, overinflation of the native lung was defined based on a qualitative assessment, such as radiological mediastinal shift, and so we had to set a novel quantitative indicator. We think that the recipient with increased rate of change in the N/T in the present study would have overinflated native lung and suffer from respiratory failure in the future. To support the clinical importance of the N/T ratio, the long-term observation and accumulation of cases will be necessary in the future. Second, this study is subject to the bias of a retrospective study because we excluded CLAD cases from this study. It may be difficult to differentiate disorders of the transplanted lung such as CLAD from the native lung overinflation when %FEV1 decreases after SLT for LAM.

The ISHLT report in 2014 described 138 LAM patients who received SLT out of a cumulative number of 15,321 cases [1]. The present study is of marked significance as it evaluated 25 patients who received SLT for LAM in a single institute. In Japan, SLT is an important surgical procedure because of the chronic shortage of donors. However, this study is limited by the fact that it was a single-center, retrospective study involving a small group of patients.

In conclusion, the rate of change in the N/T ratio occurs in some recipients of SLT for LAM during the long term and it may affect post-transplant pulmonary function.

## Supporting information

S1 Table Surgical and postoperative characteristics. (DOCX)

S2 Table Pulmonary function test, 6-minute walking distance and arterial blood gas analysis after single lung transplantation. (DOCX)

S1 Fig Correlations between native lung volume/total lung volume ratio and diffusion capacity of the lung for carbon monoxide %predicted. (A) There was no significant correlation between native lung volume/total lung volume (N/T) ratio and diffusion capacity of the lung for carbon monoxide %predicted (%DLco) at 1 year after single lung transplantation (SLT). (B) N/T ratio did not show a significant correlation with %DLco at 5 years after SLT.
(TIFF)

S2 Fig Correlations between native lung volume/total lung volume ratio and alveolar–arterial gradient. (A) There was no significant correlation between native lung volume/total lung volume (N/T) ratio and alveolar—arterial gradient (A-aDO_2_) at 1 year after single lung transplantation (SLT). (B) N/T ratio did not show a significant correlation with A-aDO_2_ at 5 years after SLT.
(TIFF)

S3 Fig Correlations between native lung volume/total lung volume ratio and 6-minute walking distance. (A) There was no significant correlation between native lung volume/total lung volume (N/T) ratio and 6-minute walking distance (6MWD) at 1 year after single lung transplantation (SLT). (B) N/T ratio did not show a significant correlation with 6MWD at 5 years after SLT.
(TIFF)

S4 Fig Correlations between rate of change in native lung volume/total lung volume ratio and change in forced vital capacity %predicted. The rate of change in the native lung volume/total lung volume (N/T) did not show a significant correlation with the rate of change in forced vital capacity %predicted (%FVC) at 5 year after single lung transplantation (SLT).
(TIFF)

S5 Fig Correlations between rate of change in native lung volume/total lung volume ratio and change in diffusion capacity of the lung for carbon monoxide %predicted. The rate of change in the native lung volume/total lung volume (N/T) did not show a significant correlation with the rate of change in diffusion capacity of the lung for carbon monoxide %predicted (%DLco) at 5 year after single lung transplantation (SLT).
(TIFF)

S6 Fig Correlations between rate of change in native lung volume/total lung volume ratio and change in diffusion capacity of the lung for carbon monoxide/alveolar volume %predicted. The rate of change in the native lung volume/total lung volume (N/T) did not show a significant correlation with the rate of change in diffusion capacity of the lung for carbon monoxide/alveolar volume %predicted (%DLco/VA) at 5 year after single lung transplantation (SLT).
(TIFF)

S7 Fig Correlations between rate of change in low attenuation volume and change in forced expiratory volume in 1 second %predicted. The rate of change in low attenuation volume (LAV) did not show a significant correlation with the rate of change in forced expiratory volume in 1 second %predicted (%FEV1) at 5 year after single lung transplantation (SLT).
(TIFF)

S8 Fig Correlations between rate of change in low attenuation volume and change in forced vital capacity %predicted. The rate of change in low attenuation volume (LAV) did not show a significant correlation with the rate of change in forced vital capacity %predicted (%FVC) at 5 year after single lung transplantation (SLT).
(TIFF)

S9 Fig Correlations between rate of change in low attenuation volume and change in diffusion capacity of the lung for carbon monoxide %predicted. The rate of change in low attenuation volume (LAV) did not show a significant correlation with the rate of change in diffusion capacity of the lung for carbon monoxide %predicted (%DLco) at 5 year after single lung transplantation (SLT).
(TIFF)

S10 Fig Correlations between rate of change in low attenuation volume and change in diffusion capacity of the lung for carbon monoxide/alveolar volume %predicted. The rate of change in low attenuation volume (LAV) did not show a significant correlation with the rate of change in diffusion capacity of the lung for carbon monoxide/alveolar volume %predicted (%DLco/VA) at 5 year after single lung transplantation (SLT).
(TIFF)

S1 Dataset “Values-for-Tables-and-Figs”. This file contains the full underlying data set for all Tables and Figs.
(XLSX)

## References

[1] Lund LH, Edwards LB, Kucheryavaya AY, Benden C, Christie JD, Dipchand AI, et al The registry of the international society for heart and lung transplantation: Thirty-first official adult heart transplant report—2014; Focus theme: Retransplantation. J Hear Lung Transplant. 2014;33:996–1008. 10.1016/j.healun.2014.08.003 25242124

[2] Miyoshi R, Chen-Yoshikawa TF, Hijiya K, Motoyama H, Aoyama A, Menju T, et al Significance of single lung transplantation in the current situation of severe donor shortage in Japan. Gen Thorac Cardiovasc Surg. 2016;64:93–7. 10.1007/s11748-015-0610-3 26620538

[3] Toronto Lung Transplant Group. Unilateral lung transplantation for pulmonary fibrosis. N Engl J Med. 1986;314:1140–5. 10.1056/NEJM198605013141802 3515192

[4] Mal H, Andreassian B, Pamela F, Duchatelle J, Rondeau E, Dubois F, et al Case Reports. 1989;797–802. 10.1164/ajrccm/140.3.797 2675707

[5] Kroshus TJ, Bolman RM, Kshettry VR. Unilateral volume reduction after single-lung transplantation for emphysema. Ann Thorac Surg. 1996;62:363–8. 8694592

[6] Wilson H, Carby M, Beddow E. Lung volume reduction surgery for native lung hyperinflation following single-lung transplantation for emphysema: Which patients. Eur J Cardio-thoracic Surg. 2012;42:410–3. 10.1093/ejcts/ezs086 22389343

[7] Perch M, Riise GC, Hogarth K, Musani AI, Springmeyer SC, Gonzalez X, et al Endoscopic treatment of native lung hyperinflation using endobronchial valves in single-lung transplant patients: A multinational experience. Clin Respir J. 2015;9:104–10. 10.1111/crj.12116 24506317

[8] Johnson SR, Cordier JF, Lazor R, Cottin V, Costabel U, Harari S, et al European Respiratory Society guidelines for the diagnosis and management of lymphangioleiomyomatosis. Eur Respir J. 2010;35:14–26. 10.1183/09031936.00076209 20044458

[9] Liu F, Ruan Z, Wang S, Lin Q. Right native lung pneumonectomy due to over inflation three years after left single lung transplantation for pulmonary lymphangioleiomyomatosis. Ann Thorac Cardiovasc Surg. 2014;20:70–3. 24088919 10.5761/atcs.cr.13-00133

[10] Barr M, Chaparro C, Corris P, Doyle R, Glanville A, Klepetko W, et al Bronchiolitis Obliterans Syndrome 2001: An Update of the Diagnostic. J Hear Lung Transpl. 2002;21:297–310. 10.1016/s1053-2498(02)00398-4 11897517

[11] Kawakami K, Iwano S, Hashimoto N, Hasegawa YNS. Evaluation of emphysema using three-dimensional computed tomography: association with postoperative complications in lung cancer patients. Nagoya J Med Sci. 2015;77:113–22. 25797976 PMC4361513

[12] Murakami J, Ueda K, Hayashi M, Kobayashi T, Kunihiro Y, Hamano K. Size-capacity mismatch in the lung: a novel predictor for complications after lung cancer surgery. J Surg Res. 2017;209:131–8. 10.1016/j.jss.2016.08.051 28032549

[13] Chen F, Kubo T, Shoji T, Fujinaga T, Bando T, Date H. Comparison of pulmonary function test and computed tomography volumetry in living lung donors. J Hear Lung Transplant. 2011;30:572–5. 10.1016/j.healun.2010.11.019 21211998

[14] Park CH, Kim TH, Lee S, Paik HC, Haam SJ. New predictive equation for lung volume using chest computed tomography for size matching in lung transplantation. Transplant Proc. 2015;47:498–503. 10.1016/j.transproceed.2014.12.025 25769597

[15] Konheim JA, Kon ZN, Pasrija C, Luo Q, Sanchez PG, Garcia JP, et al Predictive equations for lung volumes from computed tomography for size matching in pulmonary transplantation. J Thorac Cardiovasc Surg. 2016;151:1163–1169.e1. 10.1016/j.jtcvs.2015.10.051 26725712

[16] Levine SM, Anzueto A, Peters JI, Cronin T, Sako EY, Jenkinson SC, et al Medium term functional results of single-lung transplantation for endstage obstructive lung disease. Am J Respir Crit Care Med. 1994;150:398–402. 10.1164/ajrccm.150.2.8049821 8049821

[17] Haruna A, Muro S, Nakano Y, Ohara T, Hoshino Y, Ogawa E, et al CT scan findings of emphysema predict mortality in COPD. Chest. The American College of Chest Physicians; 2010;138:635–40. 10.1378/chest.09-2836 20382712

[18] Tanabe N, Muro S, Hirai T, Oguma T, Terada K, Marumo S, et al Impact of exacerbations on emphysema progression in chronic obstructive pulmonary disease. Am J Respir Crit Care Med. 2011;183:1653–9. 10.1164/rccm.201009-1535OC 21471102

[19] Matsuoka S, Yamashiro T, W R.George, Kurihara Y, Yasuo N, Hatabu H. Quantitative CT Assesment of Chronic Obstructive Pulmonary. Radiographics. 2010;30:55–66. 10.1148/rg.301095110 20083585

[20] Tobino K, Hirai T, Johkoh T, Kurihara M, Fujimoto K, Tomiyama N, et al Differentiation between Birt-Hogg-Dubé syndrome and lymphangioleiomyomatosis: Quantitative analysis of pulmonary cysts on computed tomography of the chest in 66 females. Eur J Radiol. 2012;81:1340–6. 10.1016/j.ejrad.2011.03.039 21550193

