# Supplementary material for: Correlation between the native lung volume change and postoperative pulmonary function after single lung transplantation for lymphangioleiomyomatosis: Evaluation of lung volume by three-dimensional computed tomography volumetry
Source: PLoS One. 2019 Feb 11;14(2):e0210975. doi: 10.1371/journal.pone.0210975 (PMC6370208; doi:10.1371/journal.pone.0210975)
Supplement: S1 Table — (DOCX) [file pone.0210975.s001.docx]

**S1 Table. Surgical and postoperative characteristics**

| Variable | | N = 17 |
| --- | --- | --- |
| Surgical side | |  |
|  | Right | 9 (52.9%) |
|  | Left | 8 (47.1%) |
| Mechanical ventilation period (days) | | 6.9 ± 6.5 (2–20) |
| Intensive care unit stay (days) | | 15.5 ± 9.6 (6–42) |
| Data are expressed as the mean ± standard deviation or number (%). | | |
