# Supplementary material for: Correlation between the native lung volume change and postoperative pulmonary function after single lung transplantation for lymphangioleiomyomatosis: Evaluation of lung volume by three-dimensional computed tomography volumetry
Source: PLoS One. 2019 Feb 11;14(2):e0210975. doi: 10.1371/journal.pone.0210975 (PMC6370208; doi:10.1371/journal.pone.0210975)
Supplement: S2 Table — (DOCX) [file pone.0210975.s002.docx]

**S2 Table. Pulmonary function test, 6-minute walking distance and arterial blood gas analysis after single lung transplantation**

|  | | Years after single lung transplantation | | | | | P value | |
| --- | --- | --- | --- | --- | --- | --- | --- | --- |
|  |  | 1 year  (n = 17) | 2 years  (n = 17) | 3 years  (n= 17) | 4 years  (n = 15) | 5 years  (n = 10) |  |  |
| Pulmonary function test | |  |  |  |  |  |  | |
|  | %FEV1 (%)^*^ | 58.0 ± 10.9 | 56.9 ± 12.2 | 56.0 ± 12.8 | 55.1 ± 11.2 | 50.8 ± 9.0 | 0.57 | |
|  | %FVC (%)^†^ | 73.9 ± 17.9 | 71.3 ± 17.9 | 71.5 ± 18.2 | 68.4 ± 16.5 | 61.8 ± 13.0 | 0.45 | |
|  | %DLco (%)^‡^ | 81.2 ± 16.3 | 88.4 ± 22.3 | 83.5 ± 19.1 | 85.8 ± 20.0 | 85.1 ± 23.1 | 0.88 | |
|  | %DLco/VA (%)^§^ | 82.4 ± 19.7 | 86.0 ± 15.0 | 88.2 ± 20.1 | 93.6 ± 17.8 | 98.9 ± 13.1 | 0.13 | |
|  | | 1 year  (n = 17) | 2 years  (n = 16) | 3 years  (n= 17) | 4 years  (n = 12) | 5 years  (n = 8) |  | |
| 6-minute walking distance | | 457.4 ± 78.7 | 460.4 ± 66.7 | 477.7 ± 74.4 | 488.0 ± 66.8 | 511.5 ± 53.4 | 0.37 | |
| (m) | |  |  |  |  |  |  | |
|  | | 1 year  (n = 17) | 2 years  (n = 17) | 3 years  (n= 17) | 4 years  (n = 12) | 5 years  (n = 10) |  | |
| Arterial blood gas analysis | |  |  |  |  |  |  | |
|  | PaO2 (Torr) | 83.0 ± 7.5 | 81.9 ± 8.4 | 82.0 ± 8.7 | 78.3 ± 9.3 | 80.9 ± 8.2 | 0.65 | |
|  | PaCO_2_ (Torr) | 37.7 ± 3.8 | 37.2 ± 3.1 | 37.4 ± 3.8 | 39.2 ± 2.9 | 37.8 ± 4.2 | 0.65 | |
|  | A-aDO_2_ (Torr) | 19.7 ± 7.5 | 21.3 ± 9.2 | 21.1 ± 9.8 | 22.5 ± 9.2 | 21.6 ± 7.1 | 0.93 | |
| Data are expressed as the mean ± standard deviation. ^*^ FEV1, forced expiratory volume in 1 second. ^†^ FVC, forced vital capacity. ^‡^ DLco, carbon monoxide diffusing capacity. ^§^ DLco/VA, DLco/alveolar volume. | | | | | | | |  |
